# Healthcare Workers’ Knowledge and Attitude Toward Telemedicine During the COVID-19 Pandemic: A Global Survey

**DOI:** 10.7759/cureus.30079

**Published:** 2022-10-08

**Authors:** Saman Z Naqvi, Shahzaib Ahmad, Ian C Rocha, Kimberly G. Ramos, Haseeba Javed, Fatima Yasin, Hadin D Khan, Shahzaib Farid, Aleenah Mohsin, Aiman Idrees

**Affiliations:** 1 Surgery, Betsi Cadwaladr Health Board, Rhyl, GBR; 2 Surgery, Mayo Hospital, Lahore, PAK; 3 Medicine, Centro Escolar University, Manila, PHL; 4 Medicine, Mayo Hospital, Lahore, PAK; 5 Medicine, Shalamar Medical and Dental College, Lahore, PAK

**Keywords:** telehealth, coronavirus, healthcare system, global survey, knowledge, attitude, covid 19, telemedicine

## Abstract

Introduction

Telemedicine is the utilization of communication technologies to provide healthcare services remotely. It has an increasingly pivotal role in enabling medical professionals to extend the provision of care to patients facing geographical barriers. The benefits of telemedicine have become more apparent during the coronavirus pandemic. To maximize its application, it is crucial to ascertain the understanding and attitudes of healthcare professionals toward its use. The aim of this study is to collect data and evaluate the current knowledge and perceptions of medical staff toward the use of telemedicine.

Methods

In this cross-sectional study, we conducted a global survey of 1091 healthcare workers. Data were collected through a questionnaire after an extensive literature review. Frequency, percentages, and cumulative percentages were calculated to portray the profile of the participants.

Results

Of the respondents, the majority had heard about (90.9%), witnessed (65.3%), or were familiar with (74.6%) how telemedicine is used in practice. Seventy-two point two percent (72.2%) were familiar with the tools that may be used in this technology. The familiarity with telemedicine was noted to be consistently higher in those with a medical degree and experience of less than five years. Furthermore, attitudes toward providing healthcare remotely were generally positive with 80% thinking that telemedicine reduced staff workload, 80.6% reporting that it reduces the unnecessary transportation cost, and 83% believing that it saves clinicians' time. However, 20% of respondents said that telemedicine increases staff workload and 40.5% of healthcare workers believed telemedicine threatens information confidentiality and patient privacy.

Conclusion

Although telemedicine is a novel and emerging practice in many countries, it appears to have a promising contribution to healthcare services. This is particularly important during a pandemic, as it ensures effective healthcare with the maintenance of social distancing measures. Moreover, the respondents of this study showed good knowledge and positivity in their attitude toward telemedicine.

## Introduction

The provision of effective and prompt medical care has always been at the core of decision-making by healthcare policymakers. The major hindrance to this in underdeveloped areas has been due to the delays in reaching the nearest health facility. Access to hospitals is usually difficult in these areas, both in terms of distance and financial constraints [1]. One of the emerging solutions to address this is the employment of telemedicine in delivering medical services. Telemedicine is defined by the World Health Organization (WHO) as: The provision of healthcare services at a distance with communication conducted between healthcare providers seeking clinical guidance and support from other healthcare providers or conducted between remote healthcare users seeking health services and healthcare providers [2]. Telemedicine brings to use communication technology to allow the exchange of relevant information for the purpose of diagnosing, managing, and preventing disease and illness. It also aims to further educate and train healthcare providers focusing on facilitating the health of individuals and the community [1]. The concept of telemedicine has tremendous potential as a tool for the dissemination of medical knowledge and the provision of effective medical services. However, it has faced multiple obstacles and neglect in the past. Recently, the use of telemedicine has become more acceptable and has been employed in the provision of healthcare in 125 countries [3].

Coronavirus disease 2019 (COVID-19) emerged as a viral outbreak in Wuhan, China. It was declared to be a pandemic by WHO on March 11, 2020 [4]. As the global health community struggled to grapple with the increasing number of cases and deaths, it was faced with the challenge of ensuring continuity of care. The increased workload due to COVID-19 casualties in the global population led to a general compromise in the provision of healthcare to patients suffering from non-COVID-related illnesses and unprecedented stress on healthcare systems [5]. It became critical to provide appropriate medical care for patients through a medium that ensured a reduction in physical contact and face-to-face consultations. Telemedicine thus became the most suitable platform for the safe delivery of healthcare services as well as allowing for social distancing guidelines to be maintained [2].

Despite the promising role of telemedicine, its implementation heavily relies on the active engagement of healthcare professionals. Previous research on the knowledge and attitude of healthcare professionals towards telemedicine has faced limitations such as a small sample size and data being collected from single-centered healthcare settings [6]. This study aims to explore the knowledge and attitude of healthcare professionals toward telemedicine on a larger scale, involving a diverse sample size from around the globe, to ensure that the results are representative of a larger population.

## Materials and methods

Study design and sampling

In this cross-sectional study, we conducted a global survey of 1091 healthcare workers across the globe to explore their attitudes and knowledge regarding telemedicine during the COVID-19 pandemic. Snowball sampling was used. This study included countries from Africa, Southeast Asia, Europe, the Eastern Mediterranean region, the Western Pacific region, and America. This list was based on the WHO list of regions [7]. The target population included physicians, health officers, nurses, medical laboratory scientists, pharmacists, midwives, dentists, allied health professionals, hospital staff, and medical students from health-related fields. Unregistered physicians, those who did not consent to fill out the questionnaire, and those who submitted incomplete questionnaires were excluded as well as healthcare professionals who were not actively involved in the process of patient care during the pandemic.

Study tool

Data were collected through a self-administrated questionnaire after conducting an extensive literature review. The questionnaire was completed through Google Forms that were disseminated through personalized emails and telecommunication media groups (WhatsApp) and comprised three components. The first section included sociodemographic data of the participants including their: gender, age, educational attainment, type of profession, years of working experience, salary, and residence based on the country and region (eight items). The second section encompassed questions related to the perception and knowledge of healthcare workers regarding telemedicine technology (nine items). Lastly, the third section explored the attitude of healthcare professionals towards telemedicine in terms of its benefits, applicability, and complexity of implementation (23 items). We utilized a complete pre-validated structured questionnaire developed by Biruk et al. [5].

Data analysis

In the second section, each item was assessed by a 2-point scale with a score of 0 and 1 (No=0 and Yes=1). The overall score for this section ranged from a minimum of 0 to a maximum of 9. A score of less than 50% was poor and more than 50% was considered good knowledge. The third section of the questionnaire evaluating attitude included questions on a five-point Likert scale ranging from ‘1=strongly disagree’, ‘2=disagree’, ‘3=neutral’, ‘4=agree’, and ‘5=strongly agree’. The complexity attribute was scored in the reverse direction. The score for the attributes given in the attitude section were averaged to yield the mean score for this section. A mean score of 2.6 (50%) was a negative attitude, 2.6 to 3 (50-60%) was neutral, and greater than 3 (60%) was considered as having a positive attitude toward telemedicine. The demographic characteristics were presented using frequencies, percentages, and cumulative percentages. Data were collected, labeled, and coded, after which it was analyzed using Statistical Product and Service Solutions (SPSS) version 20 (IBM Corp., Armonk, NY).

Operational definitions

In this study, telemedicine was defined per the World Health Organization's description of it being ‘The provision of healthcare services at a distance with communication conducted between healthcare providers seeking clinical guidance and support from other healthcare providers or conducted between remote healthcare users seeking health services and healthcare providers' [2]. Health professionals were defined as workers who provide service in health settings having at least a diploma certificate in a healthcare-related field.

Ethical approval

Ethical clearance was approved by the Kahuta Research Laboratories (KRL) Hospital Islamabad ethics committee. Anonymity and confidentiality were assured and informed consent was obtained from the participants.

## Results

This cross-sectional study yielded 1091 responses from across 57 countries between December 2021 and July 2022. The majority of the respondents were female (56.9%). Most of the respondents had the highest educational qualification of Bachelors (48.6%), were physicians by profession (36.9%), had a job experience of less than five years (50.1%), and a salary of less than 250 United States dollars (USD) (47.1%). The respondents were mainly residents of the Western Pacific region (71.04%) followed by the Eastern Mediterranean Region (13.75%). The age of most respondents was found to be between 18 and 39 years (81.5%) (Tables 1-8).

**Table 1:** Gender distribution of respondents

Gender	Number of respondents
Male	470
Female	621

**Table 2:** Age Groups

Age group	Number of respondents
Young Adult (age 18 to 39)	889
Middle Adult (age 40 to 59)	183
Old Adult (age 60 and above)	19

**Table 3:** Highest Educational Attainment MD: Doctor of Medicine; MBBS: Bachelor of Medicine, Bachelor of Surgery; MBChB: Bachelor of Medicine, Bachelor of Chirurgery

Educational attainment	Number of respondents
Diploma/Certificate	17
Bachelor’s Degree	530
Master’s Degree	110
Doctorate Degree	31
MD/MBBS/MBChB Degree	403

**Table 4:** Healthcare Professionals Participants grouped by type of healthcare profession

Profession	Number of respondents
Physician	403
Nurse	320
Medical Technologist	52
Pharmacist	22
Midwife	14
Health Officer	63
Allied Health Professional (Dentist, Nutritionist-Dietitian, Respiratory Therapist, Radiologic Technologist, Physiotherapist, Psychologist, Occupational Therapist)	23
Hospital Staff (Nursing Attendant, Caregiver, Paramedic, Emergency Medical Technician, Clinical Assistant)	194

**Table 5:** Years of Work Experience

Work experience	Number of respondents
Less than 5 years	547
5 to 10 years	272
More than 10 years	272

**Table 6:** Monthly Salary USD: United States dollar

Salary	Number of respondents
Less than USD 250	514
USD 250 to USD 500	172
USD 500 to USD 750	99
USD 750 to USD 1,000	100
More than USD 1,000	206

**Table 7:** Countries of Residence

Country	Number of respondents
Afghanistan	6
Argentina	1
Australia	1
Bahrain	1
Bangladesh	3
Brazil	1
Brunei	2
Cambodia	7
Canada	1
Chile	1
China	1
Cuba	4
Egypt	5
Ethiopia	1
France	2
Gambia	1
Georgia	2
Germany	5
Greece	5
Guatemala	1
India	29
Indonesia	11
Iran	3
Iraq	2
Israel	2
Italy	1
Japan	2
Kenya	5
Kuwait	1
Lithuania	1
Malaysia	73
Mexico	2
Namibia	1
Nepal	3
Netherlands	1
Nigeria	13
Pakistan	125
Peru	1
Philippines	673
Portugal	2
Qatar	4
Russia	1
Saudi Arabia	1
Sierra Leone	3
Singapore	13
Spain	3
Sri Lanka	3
Sweden	1
Tanzania	1
Thailand	6
United Arab Emirates	2
United Kingdom	9
United States	12
Uruguay	1
Venezuela	1
Vietnam	3
Zambia	25

**Table 8:** Country of Residence by Region Distribution of respondents via regions that are defined by the World Health Organization's list of regions [7]

Region	Countries	Number of respondents
Africa	Ethiopia, Gambia, Kenya, Namibia, Nigeria, Sierra Leone, Tanzania, Zambia	50
Americas	Argentina, Brazil, Canada, Chile, Cuba, Guatemala, Mexico, Peru, United States, Uruguay, Venezuela	26
South-East Asia Region	Bangladesh, India, Indonesia, Nepal, Sri Lanka, Thailand	55
Europe	France, Georgia, Germany, Greece, Israel, Italy, Lithuania, Netherlands, Portugal, Russia, Spain, Sweden, United Kingdom	35
Eastern Mediterranean	Afghanistan, Bahrain, Egypt, Iran, Iraq, Kuwait, Pakistan, Qatar, Saudi Arabia, United Arab Emirates	150
Western Pacific	Australia, Brunei, Cambodia, China, Japan, Malaysia, Philippines, Singapore, Vietnam	775

This study evaluated the knowledge and attitudes of health professionals toward telemedicine. We found most (73.69%) of the respondents reported possessing good knowledge of these telecommunication technologies. Out of these, 57.92% of health professionals were in the young adult age group, 31.71% were physicians, and 34.46% had less than five years of job experience. Seventy-eight point sixty-four percent (78.64%) reported a positive attitude toward telemedicine. Consistent with those who reported good knowledge, the majority of the participants whose feedback showed a positive attitude, were female (44.45%), from the young adult age group (63.7%), and physicians (29.69%). Furthermore, participants with bachelor’s degrees (36.29%) and health professionals with a salary of less than 250 dollars (34.46%) constituted the majority (P=0.00) (Tables 9, 10).

**Table 9:** Healthcare Professionals With Good Knowledge of Telemedicine MD: Doctor of medicine; MBBS: Bachelor of Medicine, Bachelor of Surgery; MBChB: Bachelor of Medicine, Bachelor of Chirurgery; USD: United States dollar. Respondents who reported having good knowledge of telemedicine (scoring more than 50% on the knowledge section of the questionnaire), were categorized by gender, age, educational attainment, profession, years of experience, and salary. Percentages of respondents in each category have been worked out from a total of 1091 participants. *p-values* represent the cross-tabulation of good knowledge with the corresponding demographic variables.

Characteristics	Number	Percentage (of total)	p-value
Gender			0.23
Male	355	32.54	
Female	449	41.15	
Age			0.00
Young Adult (age 18 to 39)	632	57.92	
Middle Adult (age 40 to 59)	155	14.2	
Old Adult (age 60 and above)	17	1.55	
Education			0.00
Diploma/Certificate	7	0.64	
Bachelor’s Degree	335	30.7	
Master’s Degree	91	8.34	
Doctorate Degree	25	2.29	
MD/MBBS/MBChB Degree	346	31.71	
Type of Profession			0.00
Physician	346	31.71	
Nurse	231	21.17	
Medical Technologist	32	2.93	
Pharmacist	14	1.28	
Midwife	11	1	
Health Officer	40	3.66	
Allied Health Professional	14	1.28	
Hospital Staff	116	10.63	
Years of experience			0.00
Less than 5 years	376	34.46	
5 to 10 years	200	18.33	
More than 10 years	228	20.89	
Salary			0.00
Less than USD 250	338	30.98	
USD 250 to USD 500	127	11.64	
USD 500 to USD 750	82	7.51	
USD 750 to USD 1000	74	6.78	
More than USD 1,000	183	16.77	

**Table 10:** Healthcare Professionals With a Positive Attitude Toward Telemedicine This table shows the distribution of participants who had a positive attitude toward telemedicine categorized by sociodemographic factors. *p-values* represent the cross-tabulation of positive attitudes with the corresponding demographic variables.

Characteristics	Number	Percentage (of	p-value
Gender			0.23
Male	373	34.19	
Female	485	44.45	
Age			0.65
Young Adult (age 18 to 39)	695	63.7	
Middle Adult (age 40 to 59)	146	13.38	
Old Adult (age 60 and above)	17	1.55	
Education			0.05
Diploma/Certificate	14	1.28	
Bachelor’s Degree	396	36.29	
Master’s Degree	97	8.89	
Doctorate Degree	27	2.47	
MD/MBBS/MBChB Degree	324	29.69	
Type of Profession			0.54
Physician	324	29.69	
Nurse	259	23.73	
Medical Technologist	38	3.48	
Pharmacist	16	1.46	
Midwife	10	0.91	
Health Officer	50	4.58	
Allied Health Professional	17	1.55	
Hospital Staff	144	13.19	
Years of experience			0.2
Less than 5 years	415	38.03	
5 to 10 years	222	20.34	
More than 10 years	221	20.25	
Salary			0
Less than USD 250	376	34.46	
USD 250 to USD 500	139	12.74	
USD 500 to USD 750	91	8.34	
USD 750 to USD 1000	81	7.42	
More than USD 1,000	171	15.67	

Ninety point nine percent (90.9%) of respondents had heard about telemedicine, 74.6% reported that they were aware of it, whilst 65.3% had seen a telemedicine system. Seventy-two point two percent (72.2%) said that they were familiar with tools such as telesurgery, teleconsultation, and teleconferencing. The familiarity with these systems was noted to be consistently higher for those, with job experience of less than five years (Table 11).

**Table 11:** Responses to Questions Assessing Knowledge of Telemedicine

Knowledge	Yes (%)	No (%)
Heard about telemedicine	90.9	9.1
Seen a telemedicine system	65.3	34.7
Known about telemedicine technology	74.6	25.4
Aware of telemedicine tools such as telesurgery, teleconsultation, teleconferencing	72.2	27.8
Know the effect of telemedicine on healthcare quality	76.7	23.3
Know the effect of telemedicine on reducing medical staff workload	80.0	20.0
Know about telemedicine infrastructure	46.7	53.3
Know the benefits of telemedicine in reducing unnecessary transportation costs?	80.6	19.4
Know the benefits of telemedicine in saving clinicians' time?	83.0	17.0

In the same knowledge section, 76.7% of the participants reported recognizing the effects of telemedicine on healthcare quality and 80% were aware of its effects on reducing medical staff workload. Eighty point six percent (80.6%) were knowledgeable about using this communication technology to reduce unnecessary transportation costs. Eighty-three percent (83%) said that they knew about its benefits in saving clinicians' time. However, 53.3% of the respondents reported that they were unsure about the telemedicine infrastructure (Table 11).

In the attitudes segment of the questionnaire, participants generally agreed with the recognized advantages of telemedicine and the section's overall mean score was 3.6. Compatibility within the work setting, ability to be trailed and current implementation in the hospitals showed a similar positive trend with means of 3.5, 3.5, and 3.4, respectively (Table 12). The findings concluded a positive attitude of healthcare professionals toward telemedicine.

**Table 12:** Attitude of Healthcare Professionals Toward Telemedicine

Advantages of telemedicine	Strongly disagree (%)	Disagree (%)	Neutral (%)	Agree (%)	Strongly agree (%)	Mean	
Reduce medical error	6.5	15.3	35	32. 9	10.3	3.252	
Streamlines diagnosis and treatment	6	6.1	24.5	48. 1	15.2	3.601	
Boost communication among healthcare providers	5.9	5.1	16.1	42. 5	30.3	3.859	
Telemedicine can decrease the number of visits to healthcare centers	6.6	1.7	10.2	37. 9	43.5	4.097	
Enables healthcare workers to achieve tasks more quickly	5.7	3.8	24.7	38.6	27.2	3.778	
Improve clinical decisions	5.9	10	31.8	37. 4	14.9	3.454	
Deliver a more extensive healthcare service	6.3	6.5	24	40. 6	22.5	3.662	
Total Mean Score						3.67185714	
Compatibility	Strongly disagree (%)	Disagree (%)	Neutral (%)	Agree (%)	Strongly agree (%)	Mean	
telemedicine is compatible with all aspects of work	6.8	12.9	28.8	35.7	15.8	3.408	
Telemedicine is suitable for the current setting (COVID-19)	6.2	7.9	23.2	40.5	22.2	3.646	
The use of telemedicine aligns with my working style	6.4	10.4	28	37.5	17.8	3.502	
telemedicine integrates well with the working day	6.6	10.3	28.9	36.6	17.7	3.488	
Total Mean Score						3.511	
Telemedicine trial	Strongly disagree (%)	Disagree (%)	Neutral(%)	Agree(%)	Strongly agree(%)	Mean	
Telemedicine technologies should be trialed	6.1	3.6	24.3	47.8	18.2	3.684	
I would prefer to try out telemedicine applications before using it	5.5	2.8	21.7	45	24.9	3.807	
Trailing telemedicine will be a simple task	7	16.7	31.9	32.6	11.8	3.255	
Trialing telemedicine could give important information about its effects	4.8	10.1	33.8	40.1	11.2	3.428	
Total Mean Score						3.5435	
Current implementation	Strongly disagree (%)	Disagree (%)	Neutral(%)	Agree(%)	Strongly agree(%)	Mean	
I have witnessed applications of telemedicine by other healthcare staff	6.2	8.8	30.4	38.2	16.3	3.493	
Use of telemedicine technology is evident in the hospital where I worked	8	11.9	33.2	31.3	15.6	3.346	
In my hospital, telemedicine has been utilized for a range of tasks	7.5	8.3	31	37.2	15.9	3.454	
Total Mean Score						3.431	
Disadvantages of telemedicine	Strongly disagree (%)	Disagree (%)	Neutral(%)	Agree(%)	Strongly agree(%)	Mean	
Requires a lot of mental exertion	14	34.2	28.1	11.4	12.3	2.738	
Learning to operate telemedicine is challenging	7.7	17.8	33.3	25.4	15.9	3.243	
Increases staff workload	8.8	16.9	30.2	27.6	16.5	3.261	
increases the number of staff responsibilities	12.2	37.3	28.1	10.8	11.5	2.718	
Jeopardizes information confidentiality and patient privacy	11.6	28.9	30.2	18.6	10.7	2.879	
Total Mean Score						2.9678	

Sixty-three point three percent (63.3%) showed agreement/strong agreement with the statement that telemedicine facilitates diagnosis and treatment during COVID-19. Respondents also agreed or strongly agreed that it increases communication between healthcare providers (72.8%) and reduces the number of visits to healthcare services (81.4%). Sixty-five point eight percent (65.8%) reported that telemedicine enables them to accomplish their tasks more quickly. Fifty-two point three percent (52.3%) believed that it improves clinical decisions and 63.1% said that telemedicine allows for a more extensive healthcare service (Table 12).

Furthermore, 51.5% of the participants said that telemedicine was compatible with all aspects of their job and 62.7% stated that it is a suitable means to provide health care during the current setting of the pandemic. Fifty-four point three percent (54.3%) said that telemedicine fits well with their work style. Sixty-six percent (66%) reported that it is a great opportunity to try new applications. The majority (69.9%) were of the view that telemedicine applications should be trialed before use (Table 12).

Consistent with the above findings, most respondents disagreed with the disadvantages associated with telemedicine. Forty-four point one percent (44.1%) of healthcare workers believed that it increases staff workload, whilst only 23.7% said that it requires a lot of mental exertion to learn and 41.3% said that learning to operate telemedicine is challenging. When asked about patient privacy, 40.5% of healthcare workers responded that they believed telemedicine to be a threat to the confidentiality of the information as well as patient privacy (Table 12).

## Discussion

A 2018 study conducted in North West Ethiopia reported a minority of the research participants (37.6%) displayed adequate knowledge of telemedicine [5]. Furthermore, only 14% of respondents in a French study carried out in the same year stated that they had previously practiced telemedicine and 97.9% stated they were not sufficiently trained to provide service through this technology. According to this research, 84.8% of respondents were not familiar with telemedicine regulations [8]. Conversely, our study showed good familiarity and knowledge (74.6%) of this technology. This shows how the response to providing healthcare remotely has improved after the pandemic. This is further highlighted by a study exploring the awareness and attitudes toward telemedicine in a low-resource country amidst the pandemic. It reported that 86.5% of the respondents were familiar with this technology [9].

Recent research conducted in China has also supported the use of telemedicine and reported it to be feasible, acceptable, and effective in Western China, with 76.7% of the participants agreeing that it has improved healthcare quality [10]. Furthermore, the use of remote working systems has helped in early screening as well as decreased the burnout of healthcare workers amidst the pandemic [11]. This coincides with our study showing 63.3% of the participants crediting telemedicine for increasing the efficiency of diagnosis and treatment during COVID-19. Our study also showed that the participants believed this mode of delivering medical care led to a reduced number of visits to health facilities (81.4%) during the COVID-19 period and allowed for tasks to be completed more quickly (65.8%).

However, we found that 44.1% of healthcare workers believed that it could increase staff workload. This was also found in a simulation-based study, where the group with a telepresent team leader showed a statistically significant higher score for workload. Despite this, teamwork and care provided had not been affected [12].

Forty-one point three percent (41.3%) reportedly found it challenging to learn how to operate telemedicine technologies. This issue has also been a major hindrance reported in a systematic review of barriers to telemedicine worldwide [13]. Furthermore, in another study, 42.9% of participants thought that this mode of delivering healthcare ‘disrupts the doctor-patient relationship and causes a breach of patient privacy.’ This is similar to our findings where 40.5 % of respondents had similar views [14]. However, adequate knowledge and training in telemedicine ethics and medico-legal issues can help alleviate the burden of this drawback [15].

A limitation of this study is that descriptive statistics have mainly been presented and, thus, relationships between variables could not be further explored. There is also room for evaluation of similar data from regions that have not been represented in this study, i.e., data from some regions of the world were not included in this study. Furthermore, participants were not specifically asked about their knowledge and attitude toward all the individual aspects of telemedicine and departments associated with telemedicine. Moreover, the effectiveness and feasibility of this technology may be better assessed if patients were also included in the survey.

Future studies should also focus on the use of telemedicine to address chronic conditions and psychiatric disorders. Further research into the effects of this technology at its inception can be done by focusing on countries in which it has recently been introduced. Additionally, a detailed analysis should be carried out into the different factors that may hinder the application of telemedicine.

## Conclusions

Overall, telemedicine is a novel and emerging practice within healthcare systems. In many countries, there has been a satisfactory and beneficial implementation of it. The findings of this study showed good knowledge and positivity in their views and attitude toward telemedicine. Therefore, we believe that there is an optimistic scope to introduce remote working systems worldwide after appropriate training of healthcare workers.
